# Stigmatisation and resilience in inflammatory bowel disease

**DOI:** 10.1007/s11739-019-02268-0

**Published:** 2019-12-31

**Authors:** Marco Vincenzo Lenti, Sara Cococcia, Jihane Ghorayeb, Antonio Di Sabatino, Christian P. Selinger

**Affiliations:** 1 First Department of Internal Medicine, San Matteo Hospital Foundation, University of Pavia, Pavia, Italy; 2 Department of Psychology, Zayed University, Dubai, UAE; 3 Leeds Gastroenterology Institute, Leeds Teaching Hospitals NHS Trust, Beckett Lane, Leeds, LS9 7TF UK

**Keywords:** Crohn’s disease, Quality of life, Ulcerative colitis

## Abstract

Inflammatory bowel disease, which includes Crohn’s disease and ulcerative colitis, is an immune-mediated, chronic relapsing disorder characterised by severe gastrointestinal symptoms that dramatically impair patients’ quality of life, affecting psychological, physical, sexual, and social functions. As a consequence, patients suffering from this condition may perceive social stigmatisation, which is the identification of negative attributes that distinguish a person as different and worthy of separation from the group. Stigmatisation has been widely studied in different chronic conditions, especially in mental illnesses and HIV-infected patients. There is a growing interest also for patients with inflammatory bowel disease, in which the possibility of disease flare and surgery-related issues seem to be the most important factors determining stigmatisation. Conversely, resilience represents the quality that allows one to adopt a positive attitude and good adjustments despite adverse life events. Likewise, resilience has been studied in different populations, age groups, and chronic conditions, especially mental illnesses and cancer, but little is known about this issue in patients with inflammatory bowel disease, even if this could be an interesting area of research. Resilience can be strengthened through dedicated interventions that could potentially improve the ability to cope with the disease. In this paper, we focus on the current knowledge of stigmatisation and resilience in patients with inflammatory bowel disease.

## Introduction

Inflammatory bowel disease (IBD) comprises Crohn’s disease (CD), ulcerative colitis (UC) and IBD unclassified for patients where the diagnostic distinction between CD and UC remains uncertain [1, 2]. IBD is an immune-mediated chronic condition for which currently no definitive cure is available. The natural history of IBD is characterised by periods of remission and relapse [3, 4]. While some patients may experience long periods of remission, others may experience a rather aggressive course with rapid disease progression [5]. In UC, mucosal inflammation is limited to the colon, typically extending proximally from the rectum [1]. In contrast, CD may affect any part of the gastrointestinal tract, from the mouth to the anus, and is characterised by skip lesions, and carries the risk of developing fistulas, strictures, and abscesses [2]. Our understanding of the pathophysiology leading to the development of IBD remains incomplete [6]. Most likely, there is a complex interplay of reduced intestinal barrier function, an overshooting intestinal immune response to pathogen presentation, genetic predisposition, and environmental triggers [7].

IBD is a relatively common disease with an estimated incidence of UC at 24.3 per 100,000 and CD at 12.7 per 100,000 person years in Europe [8]. Similar incidence rates are seen in other developed nations, but there has been a recent rise in its incidence in developing nations [8]. IBD can develop at any age, but peak incidence is in early adulthood, with a second peak in patients over 65 years of age. As mortality from IBD is relatively low, the prevalence of IBD is rising [9]. Medical treatment for IBD includes mesalazine therapy—for UC only—with immunosuppressive treatment for CD and UC cases not controlled by mesalazine. Advanced medical therapy of IBD includes biological treatments with anti-tumour necrosis factor α agents, anti-integrin agents, and agents against interleukin-12/23. Recently the small molecule tofacitinib has been approved for UC and many other molecules are currently in development. While the above agents are effective in inducing response and remission in IBD, at least a third of patients do not respond to medical therapy [1, 2]. There remains a high need for surgical intervention in IBD.

The symptoms of IBD can vary depending on diagnosis and phenotype, but very often include diarrhoea, bleeding per rectum, anaemia, abdominal pain and weight loss [1, 2]. In addition, patients with CD may experience fistula discharge, abscesses or bowel obstruction due to the development of strictures [2, 10], whereas severe colonic inflammation in UC may lead to toxic megacolon [11]. All the aforementioned factors largely justify the high psychological burden for patients with IBD. Anxiety and depression affect many patients with IBD [12, 13] and this is often associated with poor disease outcomes. Anxiety may be driven by physical symptoms and the fear associated with experiencing those. For example, worry about incontinence in patients with diarrhoea may lead to anxiety and social isolation. Importantly, medications such as corticosteroids, which are widely prescribed for the management of flares, may in themselves precipitate anxiety and depression [14]. The patients may also become fearful of potential disease complications leading to hospital admission, need for resection surgery or the development of IBD-related colorectal cancer [3]. These fears may often be in excess of the actual risk of such a complication. The patients may fear surgical treatment for the risk of needing a stoma and medical treatments over concerns of potentially severe complications from immunosuppressive therapy. In addition, misinformation or unchecked information sources can be associated with a higher risk of anxiety [15].

In summary, patients with IBD experience disabling physical symptoms might need life-changing surgery and a have higher level of psychological distress and illnesses. These challenges will undoubtedly also affect their social and work life. This article provides the most recent insights regarding stigmatisation that IBD patients may experience and the resilience and coping skills involved in dealing with their disease.

## Literature search strategy

**Fig. 1:**
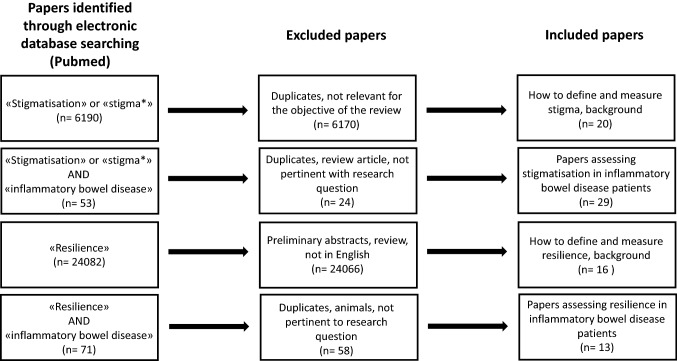
Flow diagram of paper selection that was performed through Pubmed for articles written in English. The main research question regarded the evaluation of stigmatisation and resilience in patients suffering from inflammatory bowel disease. The medical subject heading terms “Crohn’s disease” and “ulcerative colitis” were also used. More general articles dealing with how to define and how to measure stigmatisation and resilience were used to provide background for the purpose of the review

Figure 1 shows the flow diagram of paper selection. During the first week of September 2019, we searched Medline (PubMed) using the medical subject heading terms “stigmatisation”, “stigma*”, and “resilience” alone or matched with “inflammatory bowel disease”, “Crohn’s disease”, and “ulcerative colitis” for all articles published in English since inception, focusing on the research question of this review, i.e., the evaluation of stigmatisation and resilience in patients with IBD, with any of the available scales. Two authors independently searched the electronic database for papers dealing with stigmatisation (MVL, SC) and resilience (JG, MVL) in IBD patients. More than 1000 papers were found with this search strategy, the majority of which were unrelated to the subject of this review and were not considered (see Fig. 1). We, therefore, selected only landmark studies and relevant review articles for the general description of stigmatisation and resilience, focusing instead on original papers dealing with this issue in adult IBD patients. Finally, we also searched the reference lists of pivotal review articles for additional papers we judged to be relevant to this review.

## Stigmatisation: definition and how to measure it

Stigmatisation can be defined as the identification of negative attributes that distinguish a person as different and worthy of separation from the group, often leading the person losing social status and facing discrimination [16]. Social stigma is a complex and continually evolving phenomenon. It includes one’s own perception, the internalisation of negative behaviours held by other subjects, and the implementation of behaviours by other subjects [17]. Therefore, it is usually divided into three main domains. “Enacted stigma” is “stigma put into practice” and represents direct social discrimination and discriminatory experiences. “Perceived” or “felt stigma” relates to individuals feelings that other subjects have negative attitudes or negative beliefs about them and their condition. “Internalised stigma” or “self-stigmatisation” occurs when stigmatised individuals begin to conform to social mentality and to believe that stereotypes about their condition are actually true and apply to themselves [18]. A fourth recognised domain called “resistance to stigma” has been identified by some researchers using the stigma scale for mental illness (ISMI) and has been described as an adaptive response [19].

In each victim of stigmatisation, we can recognise any of the four domains of stigma and the relationship between each of them and the impact on patients’ health and well-being is well documented in the literature [20–22]. Indeed, the entity of stigmatisation depends on many factors, involving the patient, the disease, and the social context.

The stigmatisation of subjects suffering from mental illnesses has a long historical tradition, as proven by the fact that the word “stigmatisation” derives from "stigma" which is the mark that in ancient Greece was applied to slaves and criminals [23]. In the following millennia, society did not treat people who suffered from depression, autism, schizophrenia, and any other mental illness much differently to slaves and criminals. Subjects suffering from psychiatric illnesses were imprisoned, tortured, and sometimes even murdered [23]. Stigmatisation of mental illness has been identified as a critical health problem. Individuals who suffer the effects of stigmatisation due to their mental illness have a reduced life expectancy, not only due to the increase in suicides or self-harm, but due to a reduction in their physical health [24]. Such reduction is not only the consequence of both the side effects of medicines and lifestyle, but also of limited accessibility to the health services and an impaired general quality of life [24].

Besides mental illnesses, social stigmatisation is also associated with other chronic conditions. The most emblematic example is represented by patients suffering from HIV infection, who are often stigmatised due to the association of this disease with behavioural factors, such as intravenous drug abuse, and sexual promiscuity [25]. A meta-analysis based on 64 studies showed that higher levels of depression, reduced levels of social support, reduced levels of adherence to anti-retroviral therapy, and reduced access to the social services were found in HIV-infected patients who perceived high stigma [26]. Similarly, patients with lung cancer can be stigmatised due to the strong connection of the disease with cigarette smoking [27]. In lung cancer patients, it was shown that the perception of stigma resulted in increased severity of depression, anxiety and overall severity of cancer-related symptoms [28]. Among others, patients with epilepsy may perceive the stigma due to the symptoms and the care burden they exert on their loved ones, such as the inability to drive [29]. Patients with a functional illness, such as fibromyalgia, chronic fatigue syndrome, and irritable bowel disease (IBS), experience stigmatisation in relation to the fact that other people neither believe to the authenticity of their condition and, therefore, do not validate nor believe their experiences and symptoms exist [30].

Stigmatisation is assessed throughout the use of different scales, depending on the specific disorder. In patients with psychiatric diseases, the ISMI scale is most commonly used [19], while the HIV stigma scale [31] and the IBS perceived stigma scale (PSS) [32] are used for HIV and IBS patients, respectively. The same PSS used for IBS patients was also applied to IBD patients [17]. The PSS–IBD is a self-administered scale consisting of 20 statements, equally divided between significant others (friends, family members, spouse or partner) and health care providers (doctors, nurses, therapists, or other people who provide medical care). Each score is obtained by dividing the sum of the single item scores by the total number of answered items. Higher scores denote greater perceived stigma, with a range of 0 (never) to 4 (always) per every single statement.

## Stigmatisation in inflammatory bowel disease

IBD mainly affects young adults with symptoms ranging from abdominal pain and diarrhoea, to fever, weight loss, malnutrition, and intestinal obstruction requiring hospitalisation [33]. These conditions are clearly susceptible to stigmatisation, mainly because of their symptoms, the incorrect historical assumption of being psychosomatic conditions, and given that they may deeply affect sexual life and body image [34]. Intestinal symptoms can be detrimental to the patient, in particular in a social setting, as the *taboo* around such symptoms is still widespread in many cultures [17]. Furthermore, while today IBD aetiology places it in the field of immune-mediated diseases, in the past they were seen as psychosomatic illnesses in which one’s personality traits made him/her susceptible to its manifestations. The notion that a person could develop CD because of his/her "obsessive behaviour" could explain stigmatisation [35].

The patients with IBD frequently report problems related to stigmatisation, including being treated differently, having a stoma bag, and being a burden to others [36–38]. An American study recruiting patients online and through the Crohn's and Colitis Foundation showed that perceived stigma is a significant predictor of poor outcomes in IBD patients, playing a role in the quality of life of patients with detrimental effects similar to those seen in other chronic diseases [39]. The activity of the disease did not correlate with stigma perception. This may be due to the constant worry over potential flares rather than just over current symptoms [39].

Data regarding perceived stigma in IBD and IBS are controversial. Looper et al. did not find any significant difference in a study from 2004 [40], while a later study on a much larger cohort showed that IBS patients perceiving moderate-to-high stigma were three times more common than those with IBD [17].

There is now a substantial body of evidence regarding the importance of stigmatisation in IBD. These studies have focused on the various domains of stigmatisation, including perceived, internalised, and enacted stigma (Table 1) [17, 36, 38–64].

**Table 1:** Relevant studies exploring stigmatisation in patients with inflammatory bowel disease

Authors	Year	Country	Type of stigma	Sample size	Key points
Wyke et al.	1988	UK	Perceived, enacted	170	Most individuals disclosed IBD, most co-workers/employers were understanding, IBD led to changes in work
Drossman et al.	1989	USA	Perceived	150	Most common concerns related to surgery, energy level and body image
Salter	1990	USA	Internalised	Not clear	Ostomy allows “to control” the disease, feeling clumsy during sexual intercourse
Mayberry et al.	1992	UK	Enacted	58 (CD only)	Unemployment, CD patients more likely to conceal their disease to employers
Moody et al.	1992	UK	Enacted	53	Employers unwilling to employ an individual with IBD and to give time off to attend clinics
Mayberry	1999	UK	Enacted	195 (personnel managers)	Unwillingness to provide time to see the physician, IBD jeopardises promotions
Moody et al.	1999	UK	Perceived	64	Many students complain of teachers not being sympathetic, underachievement due to ill health
Moskovitz et al.	2000	Canada	Perceived	86	Poor social support is related to worse surgical outcomes
de Rooy et al.	2001	Canada	Perceived	241	Greater stigma perceived by elderly, females, patients with UC, and with low level of education;
Levenstein et al.	2001	Cross-national	Perceived, internalised	2002	Complications and variable disease evolution elicit concern; specific issues vary among countries
Daniel	2002	USA	Perceived, enacted, internalised	5	Impaired body image, feeling different, ashamed and worried about others thinking IBD is used for secondary gain
Krause	2003	Chile	Internalised	19	IBD as illness that invades all dimensions of life
Looper and Kirmayer	2004	Canada	Perceived	89 (51 IBD)	Higher level of perceived stigma in functional disorders vs other medical conditions (including IBD)
Finlay et al.	2006	USA	Perceived	148	Major differences across ethnic groups regarding knowledge of disease and social support
Smith et al.	2007	USA	Internalised	195 (71 with IBD)	Disgust related to low colostomy adjustment, low life satisfaction, low quality of life and to stronger feelings of stigmatisation
Simmons et al.	2007	UK	Internalised	51	Stoma acceptance, interpersonal relationship and location of the stoma were associated with adjustment
Taft et al.	2009	USA	Perceived	211	Perceived stigma affects quality of life
Voth and Sirois	2009	UK	Internalised	259	IBD self-blame is related to poorer outcomes
Taft et al.	2011	USA	Perceived	496	Greater stigma in IBS than IBD, patient outcomes more affected in stigmatised IBD patients
Dibley and Norton	2013	UK	Perceived	611	Emotional and psychological impact, feelings of stigma, limited lives, practical coping mechanisms
Czuber-Dochan et al.	2013	UK	Perceived	46	IBD-related fatigue not addressed in medical consultations
Taft et al.	2013	USA	Internalised	191	Social isolation common due to stigma
Czuber-Dochan et al.	2014	UK	Enacted	20 (healthcare professionals)	IBD-related fatigue poorly understood
Frohlich	2014	USA	Perceived	14	Feeling stigmatised by partner, healthcare professionals, and colleagues
Saunders	2014	UK	Perceived	16	Non-disclosure because of shame may lead to experiencing blame
Bernhofer et al.	2017	USA	Perceived	16	Feeling labelled as unable to tolerate pain
Rohde et al.	2018	USA	Enacted	127	Enacted stigma among college students decreases when IBD is disclosed
Gamwell et al.	2018	USA	Perceived	80	Indirect effect of perceived stigma on depressive symptoms as it impacts on social belongingness
Dibley et al.	2019	UK	Perceived, internalised	18	Kinship stigma is present in IBD patients

*CD* Crohn’s disease, *IBD* inflammatory bowel disease, *IBS* irritable bowel syndrome

Perceived stigma in IBD was evaluated first in 1989, showing how the functional impairment experienced by patients was greater in their social and psychological rather than in their physical dimensions [36]. Moreover, CD patients were affected by a higher degree of perceived stigma compared to UC patients. A cross-sectional survey of 611 UK patients with IBD revealed that concerns about how other people perceived IBD led to social withdrawal and isolation, in an effort to protect themselves from potential shame [55]. These findings were confirmed by a subsequent study showing how perceived stigma indirectly worsened depression and favoured social isolation [63]. A cross-sectional survey involving 2002 IBD patients from 8 countries showed that disease-related concerns varied considerably, probably due to differences in social and economic background [48]. The study highlighted the relevance of surgery, the possibility of having an ostomy, uncertainties and unpredictability of disease course, and treatment side effects. Additionally, patients often reported concerns about being a burden to others, of feeling dirty, and of having sexual and intimacy difficulties due to their disease [48].

Studies evaluating the effect of internalised stigma in IBD patients have shown how one-third of patients reported internalised stigma in addition to alienation and social withdrawal and how most engaged in stigma resistance behaviours [57]. Furthermore, it has been demonstrated that self-blame correlates with poor outcomes and leads IBD patients to avoid coping strategies [54]. Ostomy was shown to be associated with self-stigma due to the feeling of shame related to it [52].

Finally, the enacted stigma was evaluated in several studies, generally showing how being affected by IBD has an impact on working life, as IBD patients are more likely to encounter difficulties in finding a job and getting a promotion [43–45]. Disappointingly, 30% of work managers would not provide time off to their employee to attend outpatient clinic appointments [44, 45]. More recently, the enacted stigma was evaluated in a cohort of college students showing that better awareness of the disease reduced stigma [62].

As most of these studies were conducted in the late 1990s and early 2000, and given that only a few more studies have been published since the last systematic review dealing with this issue [34], there is an urgent need to carry out further studies assessing stigmatisation related to IBD.

## Resilience: definition and how to measure it

Studies on resilience vary in their methodology and samples [64–66]. Historically, most studies focused on difficult environmental circumstances and children's ability to thrive and withstand the risk factors in their surroundings [67, 68]

Resilience, from the Latin verb "resilire", means rebound or recoil [69]. The most commonly used description for medical purposes involves the ability to adapt well in the face of adversity [70]. Recently, Ungar recommended to standardisation of research on resilience by defining three distinct parts: (1) risk exposure, (2) desired outcome, and (3) protective factors [67]. However, resilience is a dynamic process that grows over time [65].

Resilience among patients with a chronic illness is often defined as an individual's ability to cope well in the face of disease [71]. Literature reviews on chronic illnesses and resilience revealed a paucity of articles including adults compared to children [72, 73]. However, resilience was either defined as a set of personal traits or as an outcome. In cancer patients undergoing treatment [74], higher levels of resilience were positively related to higher levels of activity and lower levels of psychological distress. In a study of the relation between self-silencing and resilience in women with HIV, higher rates of silencing were associated with lower levels of resilience [75]. Furthermore, higher levels of income, education, and employment were significantly associated with resilience. A review of 12 cross-sectional studies on resilience and chronic illness showed that resilience was both a significant predictor and outcome of recovery and quality of life in individuals living with a chronic condition [71]. Hence, resilience can be considered as a part of a patient’s clinical complexity [76].

There is a multitude of scales measuring resilience mostly unique to the sample or specific situation researched. A comprehensive review by Windle et al. concluded that of 15 original scales examined many lacked sufficient information regarding the psychometric ratings and theoretical underpinning of the scales [77]. Three scales were regarded as having more robust psychometric properties, Connor–Davidson resilience scale (CD-RISC) [78], resilience scale for adults (RSA), and the brief resilience scale (BRS) [79, 80].

The BRS contains six items of resilience with higher scores indicating higher levels of resilience [80]. The BRS assesses individuals' traits of resilience and their ability to cope with stress. It was initially tested on samples of cardiac rehabilitated and fibromyalgia patients. Similarly, the RSA measured 5 domains of resilience with 37 items, namely personal competence, personal structure, social competence, social support, and family coherence [79]. It was originally tested on a sample of psychiatric outpatients. The scale was later reduced to 33 items and used a semantic differential scale format for higher accuracy [81]. Lastly, the CD-RISC comprises 25 items, also measuring trait resilience on a five-point Likert scale [78]. Like the RSA, CD-RISC was first assessed among a sample of psychiatric patients. To this day, the CD-RISC has been translated into over 70 different languages and is by far the most widely used scale of resilience [82].

## Resilience in inflammatory bowel disease

Although IBD imposes a mental and physical toll on individuals [83], some individuals do report feeling stronger due to having IBD [84].

Most studies included in this review (Table 2) investigated psychological resilience and trait resilience that promoted the ability to bounce back from IBD-related adversity [85–97]. Some demographic characteristics found to be relevant to individuals with IBD included being optimistic, older [85], male, employed, not religious, and nulliparous [86]. Women with IBD more commonly reported resilience to be an essential determinant of health and both genders mentioned self-efficacy, social support, occupational balance, and job satisfaction as the main determinants of health [87]. Women with IBD and high resilience showed changes in brain-behavioural patterns, whereas the results were not conclusive for male participants [88]. Individuals whose onset of CD occurred later in life (after 30 years of age) and who performed complimentary activities appeared to be more resilient [86]. These findings were corroborated by Taylor et al.'s study, which compared level of physical activity, resilience, and health-related quality of life (HRQOL) among IBD participants [89]. A higher intensity of physical activity independently and significantly predicted a higher level of physical HRQOL, but not mental HRQOL. Resilience, on the other hand, was a significant and positive impact on mental HRQOL.

**Table 2:** Relevant studies exploring resilience in patients with inflammatory bowel disease

Authors	Year	Country	Measured resilience	Sample size	Key points
Sirois	2014	Canada	Trait resilience	155 (IBD)	High perception of health and high levels of resilience had greater odds of using CAM
Dur et al.	2014	Austria	Psychological resilience	15 adults (CD)	Resilience appeared to be more salient and relevant to women vs men
Kilpatrick et al.^a^	2015	USA	Psychological resilience	27 (IBD)	Female IBD with high resilience showed changes in brain-behavioural pattern
Scardillo et al.	2016	USA	Resilience as traits	45 adults (30 IBD)	Resilience was significantly higher amongst individuals who adapted well to their ostomy
Sehgal et al.^a^	2017	USA	Psychological resilience	113	Lower level of resilience was associated with anxiety and depression; higher resilience predicted higher QOL
Carlsen et al.	2017	USA	Trait resilience—predictor of adjustment	87 (30% adolescents, 62 CD)	Self-efficacy and resilience were significant predictors of transition readiness among adolescent and young adults with IBD
Melinder et al.	2017	Sweden	Psychosocial stress resilience	1799 (UC)	Low-to-moderate stress resilience in adolescence correlated with increased risk of CD and UC
Sirois and Hirsch	2017	Canada	Trait resilience	152 adults (51.7% CD)	No significant difference between resilient and thriving IBD patients on perceived social support, depressive symptoms, coping efficacy, and illness acceptance
Skrautvol and Naden	2017	Norway	Stress resilience	13 adults (7 CD)	Several themes were delineated, notably “creating resilience through integrative care”
Taylor et al.	2018	USA	Trait resilience	328 adults (145 UC)	Resilience positively and significantly associated with HRQOL
Acciari et al.	2019	Brazil	11 personal traits	104 adults (CD)	Individuals who were employed without children and males were more resilient than their counterparts; CD onset > 30 years old and individuals who had complimentary activities were more resilient
Hwang and Yu	2019	Korea	A set of quality influenced by society, relationships and psychology	90 adults (76 CD)	Negative relation between resilience and depression; resilience was not affected by clinical characteristics in UC patients; lower income, sleep disturbances, being unmarried negatively impacted resilience in CD patients
Luo et al.	2019	China	Dynamic process of resilience	15 adults (10 CD)	Necessary cognitive traits and resilience-specific coping mechanisms to deal constructively with IBD

*CAM* complementary and alternative medicine, *CD* Crohn’s disease, *IBS* irritable bowel disease, *HCs* healthy controls, *HRQOL* health-related quality of life, *UC* ulcerative colitis, *IBD* inflammatory bowel disease

^a^Abstracts/conference proceedings

Sehgal et al. found that lower levels of resilience were associated with significantly higher levels of anxiety and clinical depression [90]. Conversely, higher levels of resilience were found to predict better quality of life among IBD patients.

Higher levels of resilience predicted higher levels of adaptation to the ostomy; notably, perseverance—defined as a trait of resilience was the most reliable predictor [91]. Moreover, lower income, sleep disturbances and being unmarried negatively impacted the level of resilience and depression among CD patients with an ostomy. Resilience was not significantly affected by clinical characteristics in UC patients. Overall, there was a slightly higher resilience level among UC patients compared to CD patients [92].

Contrarily to the previous studies, Sirois and Hirsch drew a distinction and defined resilience as a set of traits that only promote the ability to recover from an illness [93]. The authors contrasted the concept of resilience with one's ability to thrive, the latter entailed growth above and beyond the recovery. The study examined illness acceptance, coping efficacy, depressive symptoms, and perceived social support differences among IBD patients who experienced loss, resilience, and thriving. At baseline, results indicated that across the four outcomes coping efficacy significantly distinguished those who thrived versus those who were resilient. 6 months later, this difference was no longer statistically significant. However, both resilient and thriving IBD groups were consistently reporting better psychological outcomes compared to the individuals experiencing loss from their illness.

Stress resilience was investigated in two studies [94, 95]. Melinder et al. examined prospectively a large cohort of young men from the general Swedish population speculating that low-stress resilience would predict the onset of IBD. Three quarters of subsequently diagnosed individuals had low to moderate levels of stress resilience. Skrautvol and Naden examined qualitatively stress resilience through integrative care [95]. The highly select interviewees dealt with IBD using complementary and alternative medicine (CAM) and dietary supplements stressing the perceived importance of individualising treatment plans and making changes in their lifestyle. These findings go in line with Sirois' findings that 46% of individuals with IBD used CAM as a complementary treatment to conventional medicine [96]. Although the magnitude of the relation was small, individuals with IBD who reported high perception of health and high levels of resilience had greater odds of using CAM.

During transition from juvenile to adult-centred care, both self-efficacy (SE) and resiliency were found to independently and significantly predict better transition [97]. In response, Carlsen et al. developed an e-health transfer concept to assess patient-reported outcomes, including self-efficacy, resilience, stress response among adolescents with IBD transitioning to healthcare [98].

Resiliency and IBD only began to be investigated during the last 5 years. In most studies, resiliency was perceived as a series of traits or psychological resilience, only one study defined resiliency as a dynamic process [85], and two others looked at stress resilience [94, 95]. There also seemed to be some disagreement on whether the definition entails to thrive or restore former health [93]. Moreover, the dominance of cross-sectional data, small size, and purposive samples, as well as the near absence of longitudinal studies, are some of the shared limitations across the reviewed articles [99].

**Fig. 2:**
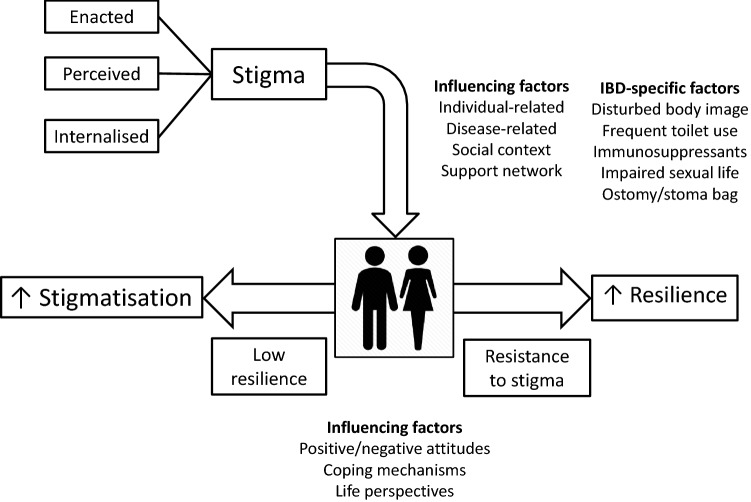
Summary of the most relevant features of stigmatisation and resilience and their influencing factors. Low resilience may favour stigmatisation, whilst resistance to stigma may strengthen resilience. Inflammatory bowel disease is burdened by a number of disease-specific issues that favour social stigmatisation and may affect resilience

Stigmatisation and resilience share many common features (Fig. 2), some of which are IBD specific, and it is reasonable to assume that they mutually influence each other, as shown for psychiatric illnesses [100] and patients living with HIV [101]. Disappointingly, only one study involving 40 community-based adult patients with self-reported IBD has investigated this issue in the IBD population so far. In that study, the authors showed that individuals who seemed more resilient were also more positive, used humour as a coping mechanism, and placed their IBD in a wider life perspective [102]. Also, stigma was more evident in patients with weak resilience, especially in those suffering from mental health disorders and in those lacking support networks.

## Future directions

Many unmet needs still exist in the IBD research agenda, including a better understanding of its physiopathology, reduction of diagnostic delays, discovery of more effective and safer drugs, optimisation of existing therapies, improving patients’ adherence to the treatment plan, improving patient’s quality of life, management of extraintestinal manifestations, and prevention of complications [103–110]. A multidimensional approach is necessary for delivering high-quality healthcare for IBD patients, but we are still far from optimal management in real life [111]. Psychosocial aspects of IBD still receive less attention than the more physical aspects of the illness. According to current evidence, stigmatisation and resilience in IBD patients are not adequately addressed in day-by-day clinical practice, even if they have a great impact in terms of quality of life and coping with the stress of a chronic illness. More holistic approaches to IBD care are required that incorporate physical, psychological, and social aspects of living with IBD.

Further research is required to better understand how stigma and resilience influence patient engagement with medical services, adherence to treatment, attitude towards healthy living, and longer-term disease outcomes. Future work to establish if and how stigmatisation can be reduced and resilience improved is urgently needed. In the authors’ opinion, the combination of better medical treatments and comprehensive approaches addressing psychosocial aspects, including stigma and resilience, will lead to a better quality of life for patients with IBD.

## Abbreviations

BRS: Brief resilience scale
CAM: Complementary and alternative medicine
CD-RISC: Connor–Davidson resilience scale
CD: Crohn’s disease
IBD: Inflammatory bowel disease
IBS: Irritable bowel disease
HRQOL: Health-related quality of life
ISMI: Stigma scale for mental illness
PSS: Perceived stigma scale
RSA: Resilience scale for adults
SE: Self-efficacy
UC: Ulcerative colitis
